# Color Doppler Patterns’ Recognition Indicative of Congenital Heart Defects at the First-Trimester Referral Scan

**DOI:** 10.3390/diagnostics15162088

**Published:** 2025-08-20

**Authors:** Valentina De Robertis, Mariachiara Bosco, Ilaria Fantasia, Claudiana Olivieri, Tiziana Fanelli, Paolo Volpe

**Affiliations:** 1 Fetal Medicine Unit, Department of Human Reproductive Medicine, 70131 Bari, Italy; derobertis_v@libero.it (V.D.R.); mariachiara.bosco@univr.it (M.B.); oliviericlaudiana@gmail.com (C.O.); fanellitiziana83@gmail.com (T.F.); pvolpe7@gmail.com (P.V.); 2 Unit of Obstetrics and Gynecology, Department of Surgery, Dentistry, Pediatrics and Gynecology, University of Verona, AOUI Verona, 37129 Verona, Italy

**Keywords:** cardiac anomalies, color doppler signs, early pregnancy, ultrasound imaging

## Abstract

**Background/Objectives**: First-trimester referral scans in high-risk pregnancies are performed by expert fetal medicine operators using an extended protocol that includes direct fetal heart assessment. This study evaluated inter-operator agreement in characterizing the four-chamber view (4CV) and three vessels and trachea view (3VTV) using color Doppler during such scans in both normal and abnormal cases. **Methods**: Two independent operators and a fetal cardiologist, all blinded to final diagnoses, retrospectively reviewed 2D images and video clips of the 4CV and 3VTV in 90 fetuses (45 with congenital heart disease [CHD] and 45 controls). The 4CV was classified into four patterns: (1) two atrioventricular (A-V) inflows of similar size, (2) one A-V inflow filling two ventricles, (3) one A-V inflow filling one ventricle, and (4) two A-V inflows with disproportion. The 3VTV was assessed for (1) normal V-sign, (2) abnormal vessel number, (3) abnormal vessel dimension, (4) abnormal spatial relationships, and (5) ductal dependence. Agreement was measured using Cohen’s Kappa. **Results**: Perfect agreement (K = 1) was seen in normal cases. In CHD cases, inter-operator and operator–cardiologist agreement was almost perfect for 4CV (K = 0.83–0.96) and substantial for 3VTV (K = 0.77–0.80). The lowest agreement occurred with ventricular disproportion in 4CV and abnormalities in vessel number and size in 3VTV. **Conclusions**: Expert operators show strong agreement in interpreting 4CV and 3VTV patterns in first-trimester scans using color Doppler. However, certain abnormalities—particularly ventricular disproportion and vessel anomalies—remain challenging to consistently interpret.

## 1. Introduction

Congenital heart diseases (CHDs) affect approximately 0.8–1.2% of live births [1] and represent the leading cause of infant mortality, accounting for 42% of infant deaths [2]. Second-trimester fetal echocardiography (FE) remains the primary diagnostic tool for CHD, playing a critical role in prognosis and management [3,4,5]. However, anticipating the prenatal detection of CHD offers several advantages, including earlier genetic workups, timely counseling, and providing couples with more time for informed decision-making [6]. Traditionally, first-trimester screening for CHD has relied on the evaluation of indirect ultrasonographic markers of cardiac anomalies, such as increased nuchal translucency (NT), tricuspid regurgitation (TR), and abnormal flow in the ductus venosus (DV) [7]. However, direct ultrasonographic evaluation of the fetal heart has emerged as a reliable method for detecting CHDs during the first-trimester scan in both low-risk [8] and high-risk [9] population. The sensitivity and specificity of this assessment vary according to the operator’s experience, population characteristics, type of cardiac abnormality, and adherence to a structured anatomical protocol [10]. Although there is ongoing debate regarding whether direct visualization of the fetal heart during the first-trimester scan should be limited to high-risk pregnancies [11,12] or extended to low-risk ones as well [13], consensus exists on the anatomical protocol to be followed [11,12,13,14]. The recently published Italian guidelines [14,15] recommend a detailed assessment of fetal anatomy during the 11 + 0 to 13 + 6 weeks scan in high-risk patients and by adopting an “extended protocol” consistent with that proposed by ISUOG guidelines [11]. This protocol includes the evaluation of the fetal heart through the four-chamber view (4CV) and three vessels and trachea view (3VTV), with color Doppler. The aim of this study was to assess the level of agreement among expert fetal medicine operators in characterizing the 4CV and 3VTV during the first-trimester referral scan in terms of color Doppler pattern identification in both normal fetal hearts and in cases with CHD.

## 2. Materials and Methods

We retrospectively reviewed images and video clips from all consecutive cases that underwent a first-trimester referral examination between January 2022 and April 2024, with a subsequent diagnosis of CHD made by a fetal cardiologist during early second- or mid-trimester fetal echocardiography and confirmed postnatally. The main indications for the referral scan were increased nuchal translucency (≥99th percentile), a family history of congenital anomalies, and/or anatomical defects suspected during the first-trimester “basic examination” in low-risk patients [14,15]. All sonographers were expert operators in first-trimester scans and certified by the Fetal Medicine Foundation (FMF). In each case, nuchal translucency (NT) thickness and additional markers were assessed, and a detailed examination of fetal anatomy was conducted following an “extended protocol” as recommended by the Italian guidelines on Ultrasound in Obstetrics and Gynecology [14,15]. First-trimester examination was performed transabdominally using a Voluson E10 (GE Medical Systems, Zipf, Tiefenbach, Austria) with sector probes C1-5-D (2–5 MHz), C2-9-D (2–9 MHz), and RAB 6D (2–8 MHz). In instances where the image quality was poor, transvaginal ultrasound evaluation was implemented by using RIC6-12D (6–12 MHz) probes (GE Medical Systems). Fetal karyotyping and/or chromosomal microarray analysis by chorionic villus sampling or amniocentesis was offered in cases at high risk for aneuploidy at the combined test or in the presence of structural defects. We subsequently identified a control group (same size) of consecutive fetuses who underwent first-trimester referral examination with normal cardiac anatomy on fetal echocardiography and a normal postnatal outcome. Cases with no satisfactory 2D images and video clips of both views were excluded from the study, as well as cases with complex CHD with the combination of multiple patterns at either the 4C view or 3VT view. For each included case, stored bidimensional (2D) and anonymized images and video clips of the 4CV and 3VTV views at color Doppler were assessed offline by two independent operators (C.O. and T.F.), both experienced in first-trimester referral scans, as well as by a fetal cardiologist (P.V.), who were blinded to each other and to the final diagnosis. The possible Color Doppler pattern variants for the 4CV were two atrioventricular (A-V) inflows of similar size, one A-V flow with filling of two ventricles, one A-V inflow with filling of one ventricle, and two A-V inflows with disproportion. The 3VTV patterns were normal V-sign, abnormal vessel number, abnormal vessel dimension, and abnormal spatial relationship of the vessels. Additionally, we decided to evaluate the presence of ductal dependence (as evidenced by a retrograde flow in one of two arches at 3VTV). Categorical data were summarized as proportions, whereas continuous variables were summarized by medians and interquartile ranges (IQRs). Maternal and fetal characteristics were compared between the two groups defined by the presence or absence of CHD. As appropriate, the Chi-square or Fisher’s exact test was used for categorical data, and the Mann–Whitney U test or *t*-test for continuous variables. Agreement between the two referral operators, as well as between each operator and the fetal cardiologist, was evaluated using Cohen’s Kappa coefficient (K). Cohen’s Kappa coefficient was also used to assess the consistency of a single operator’s assessments when reviewing the same cases at different times (intra-operator agreement) (R for Mac, ver 2024.04.0+735). The strength of agreement was interpreted according to Landis et al. [16] as <0.00 poor, 0.00–0.20 slight, 0.21–0.40 fair, 0.41–0.60 moderate, 0.61–0.80 substantial, and 0.81–1.00 almost perfect (or perfect). We also performed a simulation-based power analysis to confirm that our sample size provided maximal statistical power for detecting substantial agreement. In accordance with institutional protocols, informed consent for fetal examination and for the storage of anonymized digital images and measurement data for quality control and offline analysis for research purposes was obtained from all patients.

## 3. Results

A total of 480 high-risk pregnant patients underwent a first-trimester referral scan at our referral center between January 2022 and April 2024. Of those, 69 cases received a subsequent diagnosis of CHD made by a fetal cardiologist during early second- or mid-trimester fetal echocardiography and confirmed postnatally. Nine were excluded for poor image quality and 15 because of a complex CHD, resulting in a final cohort of 45 CHD cases. Subsequently, we identified 45 consecutive cases with a normal fetal heart. In 6 cases, images and videoclips were acquired via the transvaginal approach. The CHD cases included in this study (*n* = 45) were as follows: left and right cardiac anomalies (i.e., hypoplastic left or right heart syndrome) included those characterized by the appearance of a single ventricle (*n* = 14), atrio-ventricular septal defects (*n* = 10), conotruncal anomalies (*n* = 10), anomalies of the aortic arch (*n* = 10), and anomalies of systemic venous return (*n* = 1).

Maternal and fetal characteristics of the included cases are reported in Table 1. NT thickness [3.2 (1.8–6.4) vs. 1.6 (1.5–1.9); *p* < 0.001], DV pulsatility index [1.1 (1.0–1.8) vs. 1.0 (0.9–1.1), *p* = 0.005], and gestational age at ultrasound evaluation [13.1 (12.5–13.8) vs. 12.3 (12.0–12.6), *p* < 0.001] were significantly higher in the CHD group than in the control group. TR regurgitation [28.9% (13/45) vs. 0.0% (0/45), *p* < 0.001], reverse A wave in DV [11.1% (5/45) vs. 0.0% (0/45), *p* = 0.02], and associated extracardiac anomalies [26.6% (12/45) vs. 0.0% (0/45), *p* < 0.001] were significantly more frequent among fetuses with CHD compared to controls. No significant differences were observed for the other investigated variables.

### Inter-Operator Color Doppler Pattern Identification Agreement

**Figure 1:**
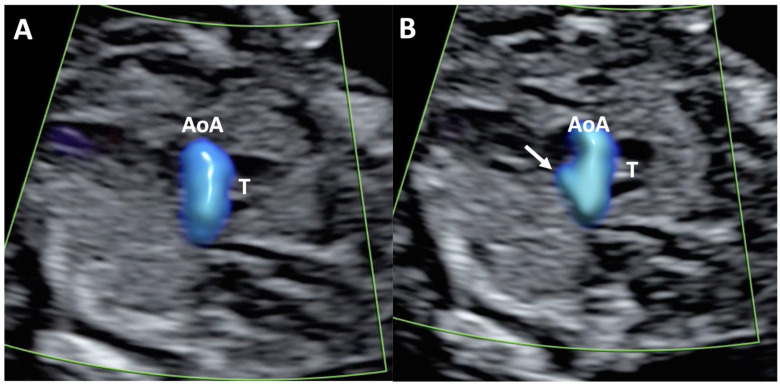
Case of disagreement in the characterization of color Doppler pattern on the 3VTV between operators 1 and 2 in a case of Ebstein anomaly at 12 + 6 weeks’ gestation. (**A**,**B**) show different frames of a videoclip of the 3VTV. (**A**) Operator 1 observed an abnormal 3VTV pattern in terms of “abnormal vessel number”; (**B**) operator 2 characterized the same case as with “abnormal vessel dimensions”. AoA, aortich arch; T, trachea; arrow, small pulmonary artery.

Overall, the analysis demonstrated an almost perfect agreement in the assessment of the total 90 included cases for both the 4CV and the 3VTV planes (K between 0.84 and 0.97) between the two operators and between each operator and the fetal cardiologist. The two operators showed a perfect agreement (K = 1) on the evaluation of the 4CV and 3VTV in the control group. Different levels of agreement were observed on the color Doppler pattern assessment in fetuses with CHD. Indeed, in this subgroup, the agreement between the operators and between each of them with the fetal cardiologist was almost perfect on the evaluation of the 4CV plane. Conversely, for the 3VTV, a lower agreement level was reported between the operators and with the fetal cardiologist (i.e., substantial agreement, K between 0.77 and 0.80). We additionally performed a sub-analysis to evaluate the inter-operator agreement in the assessment of each specific color Doppler pattern. Details regarding operators and fetal cardiologist pattern-specific agreement are displayed in Table 2. An almost perfect agreement was observed between operators 1 and 2, as well as between both operators and the fetal cardiologist, for pattern 1 (“two A-V inflows of similar size”) and pattern 3 (“one A-V inflow with filling of one ventricle”) of the 4CV, and for pattern 1 (“normal V sign”) of the 3VTV. The 4C pattern in which we observed the lowest agreement between operators and with fetal cardiologist was “two A-V inflows with ventricular disproportion” (50% agreement), while the lowest agreement on the assessment of 3VTV was reported for abnormalities “in the number” and “in the dimension” of the vessels (agreement ranging from 40% to 77.8%) (Figure 1). The overall mean K coefficient for intra-operator agreement was 0.87 with an interquartile range of 0.128, confirming good intra-operator agreement on the two views at two different times.

## 4. Discussion

Early detection of congenital anomalies is widely recognized as beneficial, providing clinical advantages for both the parents and the healthcare providers in terms of pregnancy management. Indeed, it allows more time for additional diagnostic testing and for parents to make an informed choice on pregnancy [17]. A predefined protocol for directly evaluating fetal anatomy during the first trimester across the entire population of women—including those without any risk factors for congenital anomalies screening—is recommended, as it has been shown to improve the detection rate of major structural abnormalities in early gestation [15,18,19]. Considering that most cases of CHD occur in this group, the direct evaluation of the fetal heart at the first-trimester routine scan would have the greatest impact on improving the detection rate of CHD at this gestational age. However, whether to include this assessment in the screening protocols remains debated [11,13,14,20]. Conversely, the direct study of the fetal heart is contemplated in an “extended protocol” performed during the first trimester by experienced operators [11,15]. This extended evaluation is usually part of a first-trimester referral scan offered to women at high risk of congenital anomalies [12], similar to what is offered in the second trimester to the same group of patients [21]. It includes the assessment of fetal situs, the 4CV, and the 3VTV with the aid of color Doppler mode. This approach, referred to as “simplified echocardiography” [22], has proven to be a feasible and reliable method for detecting CHD [22,23]. It differs, however, from the “early fetal echocardiography”, which is an expert examination of the fetal heart performed by the fetal cardiologist before 16 weeks’ gestation to reach the diagnosis of a specific CHD [24,25,26].

The implementation of color Doppler is essential for cardiac evaluation at early gestational age, and a correct Doppler setting represents a key aspect for the correct identification of sonographic signs of CHD [26]. Efforts to identify ultrasound patterns on the 4CV and 3VT that correlate with specific CHD have been made to support the sonographer in diagnosing cardiac anomalies in the midtrimester of pregnancy [27,28]. However, as the heart is mainly completely developed also at the time of first-trimester evaluation, these color Doppler imaging patterns can be searched and identified also at this earlier gestational age. A schematic description of the ultrasonographic abnormal findings through color Doppler patterns is particularly beneficial in the first trimester, where, given the small size of the fetal heart, the outflow tract views are difficult to obtain and interpret [29,30]. The operator’s ability to recognize and categorize these patterns is key to the correct diagnostic workup.

### 4.1. Our Results in the Contest of What Is Known

To the best of our knowledge, this is the first study that aimed to assess the ability of experienced operators in recognizing normal and abnormal color Doppler patterns on the 4CV and 3VTV of the fetal heart in the first trimester of pregnancy. We also evaluated how the operators performed compared to a fetal cardiologist, which was considered the gold standard in the evaluation of the fetal heart. We observed a perfect agreement (K = 1) between the two operators in evaluating the 4CV and 3VTV in normal cases (control group), which confirmed the high level of confidence experienced operators have in excluding the presence of CHD in normal cases. This is in line with previously published data, which showed a very high specificity (99.75%) for major CHD identification at direct first-trimester fetal heart evaluation in high-risk population [10]. However, a different agreement level was observed on the color Doppler pattern assessment in cases where a CHD was present. Indeed, in this subgroup of cases, the agreement between the operators and between each of them with the fetal cardiologist remained high (almost perfect) during 4CV plane evaluation, but it slightly dropped in the evaluation of the 3VTV. When we evaluated operators’ performance in the assessment of each specific color Doppler pattern on the 4CV, an almost perfect or substantial agreement was observed between operators and with the fetal cardiologist for all patterns, with the exception of pattern 4, which corresponded to ventricular disproportion. For this pattern, a significant discrepancy was observed in the level of agreement between operator 1 and operator 2 with the fetal cardiologist (Table 2). This inconsistency could be attributed to the limitations in subjectively perceiving the size difference between the two-color signals passing through the A-V valves at gestational ages, where the fetal dimension represents a challenge in the evaluation of anatomical structure, particularly the heart. This aspect might also account for the low level of agreement recorded in the assessment of number or dimension abnormalities on the 3VTV (patterns 2 and 3) (Table 2). Indeed, these signs are very subtle and hard to recognize and correctly interpret during the first trimester of pregnancy. This aligns with the lower detection rate reported in the literature for cardiac anomalies characterized by these abnormal findings on the 4CV and 3VTV (i.e., 4CV pattern 4 and 3VTV patterns 2 and 3) [29,30], such as coarctation of the aortic arch, pulmonary and aortic valve stenosis, and conotruncal anomalies [10]. In fact, for these groups of CHD, the reported detection rate in the literature ranges between 25% and 60% [10], which is significantly lower than the rate reported for univentricular heart, A-V septal defects, and complex cardiac defects (>60%) [10]. Operator expertise in correctly recognizing and interpreting the abnormal color Doppler signs displayed on the screen is pivotal in making the correct diagnostic process, and human error at this task has been demonstrated to be one of the most common causes of false-negative results in the first-trimester assessment of the fetal heart [26,31]. While our findings suggest that specific Color Doppler flow patterns can support the early identification of CHD, particularly in cases of univentricular heart, their diagnostic value may be limited in cases with subtle ventricular disproportions or subtle abnormalities visible only in the three vessels–trachea view. This underscores the importance of prospective studies to further validate these observations and refine their clinical applicability.

### 4.2. Strengths and Limitations

This study has several strengths. Two fetal medicine experts and a fetal cardiologist, blinded to the final diagnosis, evaluated a consistent number of cases of CHD in the first trimester of pregnancy in terms of color Doppler signs defined before study initiation, as well as an equal number of cases with normal fetal anatomy. The main limitations of this study are its retrospective design and the reliance on pre-selected stored images and videoclips, which may introduce bias and do not fully replicate real-time clinical conditions. Including only cases with high-quality 2D images and video clips introduced a selection bias, minimizing the influence of factors such as ultrasound machine settings, maternal BMI, and fetal position, which can affect fetal heart assessment in real-life clinical settings. Additionally, the need to exclude complex CHD, characterized by the coexistence of multiple abnormal 4CV and 3VTV patterns, may have introduced a potential source of bias. These factors should be considered when interpreting the study’s findings and their applicability to real-world settings. Future studies should reassess inter-operator agreement in real-life cohorts.

## 5. Conclusions

Our study demonstrated that a fetal medicine expert can reliably identify color Doppler patterns indicative of normal and abnormal cardiac anatomy. However, this performance lowered when subtle color Doppler signs of CHD were present, mainly on the 3VTV.

## Figures and Tables

**Table 1:** Maternal and fetal characteristics of the study population.

	Total (*n*: 90)	CHD (*n*: 45)	Controls (*n*: 45)	*p*-Value
Maternal age (years)	35 (32–38)	36 (33–40)	34 (29–37)	0.06
BMI (kg/m^2^)	24.4 (21.3–27.4)	26.0 (22.0–27.4)	23.9 (20.7–26.4)	0.11
GA at diagnosis (weeks)	12.6 (12.3–13.05)	13.1 (12.5–13.8)	12.3 (12.0–12.6)	<0.001
DV pulsatility index	1.1 (0.9–1.2)	1.1 (1.0–1.8)	1.0 (0.9–1.1)	0.005
CRL (mm)	63.0 (60.4–68.2)	62.0 (58.0–67.6)	64.8 (61.6–68.3)	0.16
NT (mm)	1.8 (1.5–2.4)	3.2 (1.8–6.4)	1.6 (1.5–1.9)	<0.001
PAPPA MoM	0.9 (0.6–1.2)	0.8 (0.4–1.3)	0.9 (0.7–1.2)	0.15
ART	2.2% (2/90)	2.2% (1/45)	2.2% (1/45)	1.0
TR	14.4% (13/90)	28.9% (13/45)	0.0% (0/45)	<0.001
Reverse A wave in DV	5.5% (5/90)	11.1% (5/45)	0.0% (0/45)	0.02
Associated extracardiac anomalies	13.3% (12/90)	26.6% (12/45)	0.0% (0/45)	<0.001

ART, assisted reproductive technology; BMI, body mass index; CRL, crown-rump length; DV, ductus venosus; GA, gestational age; NT, nuchal translucency; PAPPA, pregnancy-associated plasma protein A; TR, tricuspid regurgitation.

**Table 2:** Pattern-specific operators’ agreement.

US View	Color Doppler Pattern		OP 1 vs. OP2	OP 1 vs. FC	OP 2 vs. FC
4C—pattern 1	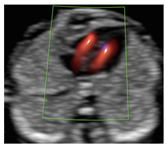	Two atrioventricular (A-V) inflows of similar size	82.6% agreement (19 agreed cases out of 23 total cases)	100.0% agreement (22 agreed cases out of 22 total cases)	82.6% agreement (19 agreed cases out of 23 total cases)
4C—pattern 2	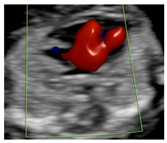	One A-V flow with filling of two ventricles	88.9% agreement (8 agreed cases out of 9 total cases)	90.0% agreement (9 agreed cases out of 10 total cases)	80.0% agreement (8 agreed cases out of 10 total cases)
4C—pattern 3	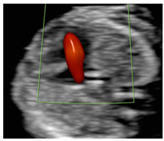	One A-V inflow with filling of one ventricle	100.0% agreement (11 agreed cases out of 11 total cases)	90.9% agreement (10 agreed cases out of 11 total cases)	90.9% agreement (10 agreed cases out of 11 total cases)
4C—pattern 4	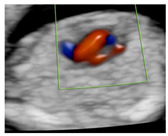	Two A-V inflows with disproportion	50.0% agreement (3 agreed cases out of 6 total cases)	100.0% agreement (3 agreed cases out of 3 total cases)	50.0% agreement (3 agreed cases out of 6 total cases)
3VT—pattern 1	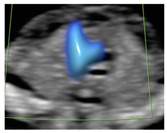	Normal V-sign	90.9% agreement (10 agreed cases out of 11 total cases)	90.9% agreement (10 agreed cases out of 11 total cases)	100.0% agreement (11 agreed cases out of 11 total cases)
3VT—pattern 2	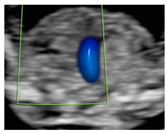	Abnormality in vessel number	60.0% agreement (6 agreed cases out of 10 total cases)	40.0% agreement (4 agreed cases out of 10 total cases)	40.0% agreement (4 agreed cases out of 10 total cases)
3VT—pattern 3	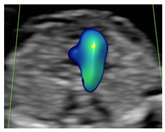	Abnormality in the vessel dimension	44.4% agreement (4 agreed cases out of 9 total cases)	77.8% agreement (7 agreed cases out of 9 total cases)	44.4% agreement (4 agreed cases out of 9 total cases)
3VT—pattern 4	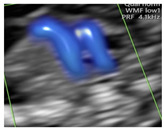	Abnormality in the spatial relationship of the vessels	80.0% agreement (12 agreed cases out of 15 total cases)	85.7% agreement (12 agreed cases out of 14 total cases)	92.9% agreement (13 agreed cases out of 14 total cases)
3VT—pattern 5	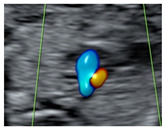	Ductal dependence (aortic or ductal arch reverse flow)	62.5% agreement (5 agreed cases out of 8 total cases)	62.5% agreement (5 agreed cases out of 8 total cases)	55.6% agreement (5 agreed cases out of 9 total cases)

US, ultrasound; OP, operator; FC, fetal cardiologist; 4C, four chambers; A-V, atrioventricular; 3VT, three vessels–trachea.

## Data Availability

The data presented in this study are available on request from the corresponding author due to the privacy policies of the hospitals involved.
